# Insight Into Interactions of Thermoacidophilic Archaea With Elemental Sulfur: Biofilm Dynamics and EPS Analysis

**DOI:** 10.3389/fmicb.2019.00896

**Published:** 2019-05-10

**Authors:** Ruiyong Zhang, Thomas R. Neu, Qian Li, Véronique Blanchard, Yutong Zhang, Axel Schippers, Wolfgang Sand

**Affiliations:** ^1^Federal Institute for Geosciences and Natural Resources (BGR), Hanover, Germany; ^2^Biofilm Centre, Universität Duisburg-Essen, Essen, Germany; ^3^Department of River Ecology, Helmholtz Centre for Environmental Research-UFZ, Magdeburg, Germany; ^4^Key Laboratory for Water Quality and Conservation of the Pearl River Delta, Ministry of Education, School of Environmental Science and Engineering, Guangzhou University, Guangzhou, China; ^5^Institute of Laboratory Medicine, Clinical Chemistry and Pathobiochemistry, Charité – Universitätsmedizin Berlin, Corporate Member of Freie Universität Berlin, Humboldt-Universität zu Berlin, and Berlin Institute of Health, Berlin, Germany; ^6^College of Environmental Science and Engineering, Donghua University, Shanghai, China; ^7^TU Bergakademie Freiberg, Freiberg, Germany

**Keywords:** attachment, biofilm, EPS, thermophile, elemental sulfur, fluorescence microscopy

## Abstract

Biooxidation of reduced inorganic sulfur compounds (RISCs) by thermoacidophiles is of particular interest for the biomining industry and for environmental issues, e.g., formation of acid mine drainage (AMD). Up to now, interfacial interactions of acidophiles with elemental sulfur as well as the mechanisms of sulfur oxidation by acidophiles, especially thermoacidophiles, are not yet fully clear. This work focused on how a crenarchaeal isolate *Acidianus* sp. DSM 29099 interacts with elemental sulfur. Analysis by Confocal laser scanning microscopy (CLSM) and Atomic force microscopy (AFM) in combination with Epifluorescence microscopy (EFM) shows that biofilms on elemental sulfur are characterized by single colonies and a monolayer in first stage and later on 3-D structures with a diameter of up to 100 μm. The analysis of extracellular polymeric substances (EPS) by a non-destructive lectin approach (fluorescence lectin-barcoding analysis) using several fluorochromes shows that intial attachment was featured by footprints rich in biofilm cells that were embedded in an EPS matrix consisting of various glycoconjugates. Wet chemistry data indicate that carbohydrates, proteins, lipids and uronic acids are the main components. Attenuated reflectance (ATR)-Fourier transformation infrared spectroscopy (FTIR) and high-performance anion exchange chromatography with pulsed amperometric detection (HPAE-PAD) indicate glucose and mannose as the main monosaccharides in EPS polysaccharides. EPS composition as well as sugar types in EPS vary according to substrate (sulfur or tetrathionate) and lifestyle (biofilms and planktonic cells). This study provides information on the building blocks/make up as well as dynamics of biofilms of thermoacidophilic archaea in extremely acidic environments.

## Introduction

Bioleaching is the process of dissolution of metal sulfides (MS) by acidophilic microorganisms. It is widely used at industrial scale for recovery of valuable metals, e.g., copper, zinc (Schippers et al., 2014). Current global production consists of 15% copper, 5% gold and smaller amounts of other metals using biomining technologies (Johnson, 2014). Unintended metal sulfide dissolution results in serious environmental problems such as acid mine drainage (AMD) or acid rock drainage (ARD) (Hallberg, 2010). Various sulfur intermediates are formed through either thiosulfate or polysulfide pathways during MS dissolution (Vera et al., 2013). As one of the major intermediates in the polysulfide pathway (Sand et al., 2001), elemental sulfur (S^0^) may form passive layers, which are chemically inert and may hinder further chemical reactions on the solid’s surface, thus preventing further MS dissolution (Rodrıguez et al., 2003; Tanne and Schippers, 2017). Subsequently, metal leaching is hindered. Sulfur-metabolizing acidophilic bacteria and archaea utilize various reduced inorganic sulfur compounds (RISCs) occurring naturally (Dopson and Johnson, 2012). Biooxidation of RISCs prevents the accumulation of sulfur-rich layers on MS surfaces, thus leading to an increased leaching rate and efficiency. Meanwhile, the oxidation of RISCs (e.g., S^0^) generates protons (Eq. 1), which keep most of the metals released from the degraded MS as ions in solution (Johnson, 2014).

$$S^{0}+H_{2}O+1.5O_{2}\to H_{2}{SO}_{4}$$

S^0^ has been often used as a source of lixiviant to generate sulfuric acid for bioleaching of valuable metals from mine tailings (Marrero et al., 2015) and electronic waste (Hong and Valix, 2014).

S^0^ has a poor solubility in water (<5 μg/L) under standard conditions (Boulegue, 1978). It was shown that sulfur-reducing microorganisms like *Acidilobus sulfurireducens* 18D70 and *Desulfurella amilsii* do not need physical contact with S^0^ for growth (Boyd and Druschel, 2013; Florentino et al., 2018). The anoxygenic phototroph *Chlorobaculum (C.) tepidum* degrades S^0^ via contact and non-contact, however, initiation of sulfur degradation has to occur in direct contact (Hanson et al., 2016), since the unattached cells metabolize S^0^ via soluble compounds, e.g., polysulfides produced by the attached cells (Marnocha et al., 2016). In case of acidophilic sulfur-oxidizing microorganisms, a direct contact between cells and S^0^ and the excretion of surface-active compounds like phospholipids seems to be necessary prior to intracellular sulfur oxidation (Schaeffer et al., 1963; Arredondo et al., 1994). In the genera of *Acidithiobacillus* and *Acidiphilium*, extracellular elemental sulfur is mobilized by thiol groups of special outer-membrane proteins and transported into the periplasmic space as persulfide sulfur (Rohwerder and Sand, 2003). Attachment of microorganisms to a solid surface is a complex process involving numerous factors such as hydrophobicity, cell surface structures, substratum properties, and others. Microbial cells can be adhered reversibly or irreversibly to a surface before firm attachment (van Loosdrecht et al., 1990). Extracellular polymeric substances (EPS), contributing significantly to the organization and structural integrity of the communities, play a crucial role in attachment and biofilm formation of cells to material surfaces. In general, EPS are proteins, carbohydrates, lipids and nucleic acids plus metal ions (Flemming and Wingender, 2010). They are subdivided into two types: capsular EPS, which are tightly bound to cells, while colloidal EPS are weakly bound to cells and easily released into the medium (Sheng et al., 2010).

The EPS concept and their analysis in bioleaching were first introduced by Sand and colleagues for acidophilic bacteria such as *Acidithiobacillus* (Sand et al., 1995; Gehrke et al., 1998), since these were thought to be the most important microorganisms in bioleaching and, therefore, were in focus. Later, researchers noticed that archaea may contribute as much as their counterparts (bacteria) in the bioleaching processes (Edwards et al., 2000b; Wheaton et al., 2015). Archaea are often the leaching organisms of choice in technical applications of bioleaching like reactor leaching in the temperature range of 50 up to 90°C. They also play a role in (self-heating) waste rock piles and (slag) heaps (Castro et al., 2019). To date there is little information available on the interaction between thermoacidophilic archaea and their natural substrates such as S^0^ and especially EPS of archaea and their biofilms in extreme environments (van Wolferen et al., 2018). *Sulfolobus* showed preferential attachment to defect sites on S^0^ (Weiss, 1973). Extracellular proteins and extracellular DNA (eDNA) were present in the EPS matrix of *Sulfolobus (S.) metallicus* DSM 6482^T^ grown on S^0^. Sugar monomers like mannose, glucose, galactose, *N*-acetylgalactosamine (GalNAc), and *N*-acetylglucosamine (GlcNAc) were detected. In addition, cells were found to be embedded in a flexible matrix (Zhang et al., 2015b).

Several physical and chemical methods have been applied to analyze EPS and biofilm composition. Imaging approaches such as Raman microscopy and scanning transmission X-ray microscopy (STXM) or fluorescence confocal laser scanning microscopy (CLSM) are available for biofilm matrix analysis (Xia et al., 2013). Among these, the only suitable *in situ* approach to analyze the EPS of biofilms is CLSM combined with fluorescence lectin-binding analysis (FLBA) and fluorescence lectin bar-coding (FLBC) (Neu and Lawrence, 2014; Neu and Kuhlicke, 2017). Besides, attenuated total reflectance (ATR)-Fourier transformation infrared spectroscopy (FTIR) is cell-surface-sensitive (Devasia et al., 1993) and has been used successfully as a rapid non-destructive technique to characterize the surface composition of different microbial systems (Jiang et al., 2004; Liu et al., 2018).

Nine *Acidianus* species have been isolated and described from different solfatara fields and marine hydrothermal systems. All nine species are thermophiles (optimal growth temperature ≥65°C), facultative anaerobes and sulfur- and iron-oxidizing chemolithoautotrophs (Wheaton et al., 2015). The presence of *A. brierleyi* greatly accelerates the leaching of chalcopyrite concentrates. It was found to be the dominant thermophile in bioleaching communities (Dinkla et al., 2009).

In this study, research questions related to a thermoacidophilic archaeon have been addressed. *Acidianus* sp. strain DSM 29099 (GenBank accession no. KJ921703) was isolated from a hot spring at Copahue Volcano (Neuquén, Argentina) (Giaveno et al., 2013; Zhang et al., 2019). How does this thermophilic archaeon attach to S^0^ and does it form a biofilm on this substrate? Does it produce EPS on S^0^? Is the attachment method of this archaeon similar to that described for bacterial species? To answer these questions several advanced microscopic techniques, e.g., AFM, CLSM, EFM, and scanning electron microscopy (SEM) were applied to visualize biofilm development on S^0^. Wet chemistry, ATR-FTIR, and HAPE-PAC were used to analyze the EPS composition after growth on different substrates.

## Materials and Methods

### Medium and Cultivation

Cells of *Acidianus* sp. DSM 29099 were cultivated in Mackintosh medium (Mac) (Mackintosh, 1978), containing 10 g/L S^0^ powder (Roth #9304, Germany), 2 g/L potassium tetrathionate (PT) or 20 g/L iron(II) sulfate at 65°C with agitation and aeration. 0.2 g/L yeast extract (YE) was added where stated. The type strain, the thermoacidophilic bioleaching archaeon *S. metallicus* DSM 6482^T^, was used in some cases for comparison. Initial pH 3.5 or 1.7 was used for PT/S^0^ or iron medium, respectively. Planktonic cells were counted directly by a light microscope (Leica DM3000) in phase contrast mode (400× magnification) with a Thoma Chamber (Hecht-Assistent). pH was measured with a digital pH meter (Model pH 537, WTW). Sulfate production due to microbial sulfur oxidation (Eq. 1) was determined by indirect atomic absorption spectroscopy (Dunk et al., 1969). For attachment and biofilm formation cells in the late exponential phase grown on S^0^ were harvested by centrifugation at 8000 rpm for 15 min. Sulfur cubes and prills (Supplementary Figure S1) were prepared as previously described (Zhang et al., 2015a). Mobility assay was performed on 0.15% Gellan gum plates supplemented with 0.001% YE.

### Hydrophobicity Test

For measuring cell surface hydrophobicity a variation of the microbial adhesion to hydrocarbons (MATH) technique was used (Rosenberg et al., 1980). n-dodecane was used as the apolar solvent (Vernhet and Bellon-Fontaine, 1995). Briefly, the mid logarithmic phase cultures were centrifuged at 8000 *g* for 15 min. Cells were washed twice with Mac medium and resuspended in this medium to an optical density of 0.3 measured at 600 nm. 2 mL of the suspension was added to a test tube (1.7 cm diameter, 15 cm length) with 0.5 mL n-dodecane and mixed on a vortex mixer at full speed for 2 min. After 15 min of settling, the aqueous phase (cell suspension) was carefully removed. The percentage of adherent cells could be ascertained by the decrease in absorbance of the aqueous phase following the assay, as compared to the absorbance of the original bacterial suspension. For comparison, chloroform was used as the acidic solvent (electron acceptor) as it has negligible basic character (Snyder, 1974). However, our preliminary results indicated that chloroform was not suitable, and the results of the hydrophobicity test are only reported for n-dodecane.

### Attachment Tests

For cell attachment assays, 10 g of sterile sulfur prills were incubated with cells of *Acidianus* sp. DSM 29099 in 50 mL Mac medium (initial cell concentration 1–2 × 10^8^ cells/mL) while shaking at 120 rpm. A control without sulfur prills was used to evaluate the non-specific cell attachment. Planktonic cells were counted using a Thoma chamber at different time intervals within 6 h. The number of attached cells was calculated by subtracting the number of planktonic cells from the initial one (Harneit et al., 2006). Experiments were done in triplicates.

### Scanning Electron Microscopy (SEM)

Planktonic cells grown under different conditions were recovered on a 0.2 μm pore-size filter. Sulfur cubes were washed twice with sterile Mac medium without any fixation. Samples were dehydrated successively with a graded acetone (ASC reagent, Sigma-Aldrich) series and stored overnight at 4°C in 90% acetone. The samples were dried by critical-point drying and coated with graphite and gold. Specimens were examined with a JEOL JSM-6330F microscope, FE-SEM at 10 kV to determine the cell spatial distribution as well as sulfur surface topography.

### Confocal Laser Scanning Microscopy (CLSM)

Cells were visualized by nucleic acid stains Syto 9, SYBR Green, DDAO (7-hydroxy-9H-1,3-dichloro-9,9-dimethylacridin-2-one), lipophilic stain FM4-64, or protein stain Sypro orange. The nucleic acid stains can be used to show the cell distribution on sulfur surfaces. Protein specific binding stain Sypro Orange was applied to visualize the proteinaceous compounds in biofilms. FM dyes are lipophilic probes, which are widely used to image synaptic vesicle exocytosis and endocytosis as well as biofilm formation by *Mycoplasma* (Gaffield and Betz, 2006). A lipid binding dye FM4-64 was used to trace hydrophobic compounds inside biofilms. Biofilms on sulfur surfaces were stained with lectins conjugated with Alexa 488 (AAL-A488) or tetramethyl rhodamine isothiocyanate (TRITC) in a coverwell chamber of 20 mm in diameter and 0.5 mm in depth (Invitrogen) or a coverwell chamber 20 mm in diameter with 0.5 mm in depth or a 5 cm Petri dish. Lectins are proteins or glycoproteins capable of binding reversibly and specifically to carbohydrates without altering their structures. The details of these stains can be found in the previous report (Zhang et al., 2015a). Samples were neutralized with filter-sterilized tap water and incubated with 0.1 mg/mL lectins for 20 min at room temperature. Afterwards, stained samples were washed three times with filter-sterilized tap water in order to remove the un-bound lectins. Direct light exposure was avoided. Counter staining was done in a coverwell chamber of 20 mm in diameter and 0.5 mm in depth (Invitrogen). Counter-stained samples were directly observed using confocal laser scanning microscopy (CLSM) without any further treatment. Examination of stained biofilms was performed by CLSM using a TCS SP5X, controlled by the LASAF 2.4.1 build 6384 (Leica, Heidelberg, Germany). The system was equipped with an upright microscope and a super continuum light source (470–670 nm) as well as a 405 nm laser diode. Images were collected with a 63× water immersion lens with a numerical aperture (NA) of 1.2 and a 63× water immersible lens with an NA of 0.9.

### Atomic Force Microscopy and Epifluorescence Microscopy

After incubation with cells of *Acidianus* sp. DSM 29099, sulfur cubes were rinsed with sterile Mac medium and deionized water. Cells attached on S^0^ and their EPS glycoconjugates were stained by Syto 9 and fluorescently labeled with Con A, respectively. Stained samples were dried at room temperature and visualized by an epifluorescence microscope (Zeiss, Germany) and AFM (BioMaterial^TM^ Workstation, JPK Instruments) for the investigation of cell morphology and distribution of cells and EPS on surfaces of S^0^. AFM studies were conducted in contact mode in air or water as previously described (Mangold et al., 2008; Li and Sand, 2017).

### EPS Extraction and Characterization

Extracellular polymeric substances fractions including colloidal EPS, capsular EPS and EPS from biofilm/sessile cells were extracted by EDTA according to the previous reports (Castro et al., 2014). Briefly, planktonic cells and sulfur particles with biofilm cells were separated by filtration through sterile Whatman filter paper. Cells were collected afterwards by centrifugation at 8000 rpm (11300 *g*) for 15 min. Cell pellets were washed by Mac medium and freeze-dried (ALPHA 2-4 LSC, -80°C). The supernatant was filtered further through polycarbonate filters (GTTB, ∅2.5 cm, 0.2 μm pore size, Millipore^®^) to remove whole cells. These cell-free supernatants (containing “colloidal EPS”) were dialyzed using a cellulose membrane (cutoff 3.5 KDa) against deionized water at 4°C for 48 h. Dialyzed colloidal EPS solutions were further freeze-dried. Capsular EPS were extracted from cell pellets using 20 mM EDTA as previously described (Castro et al., 2014). Sulfur particles were manually milled in a mortar and incubated with 20 mM EDTA at 4°C and shaking at 180 rpm for 4 h to extract EPS from biofilm cells. The extraction was repeated three times and the resulting solutions were centrifuged, filtered and dialyzed as described above. Carbohydrate determination was done by the phenol-sulfuric acid method with glucose as standard (Dubois et al., 1956). Uronic acids were estimated using glucuronic acid as standard (Pronk and Johnson, 1992). Protein concentration was analyzed with bovine serum albumin (BSA) as standard (Bradford, 1976). Lipids were quantified by the vanillin method (van Handel, 1985). Cell lysis was estimated by measuring glucose-6-phosphate dehydrogenase (G6PDH) activity (Ng and Dawes, 1973). Briefly, 10 mL of cell suspensions before EPS extraction were disrupted by sonication (five intervals of 7 min each with 3 min breaks between intervals at 40 W). The resulting lysate and EPS samples were measured for their G6PDH activities. The percentage of G6PDH activity in EPS samples and in lysate was calculated and reflected the cell lysis during EPS extraction procedure.

For sugar monomers, EPS samples were hydrolyzed in 2 N trifluoroacetic acid for 4 h at 100°C. After evaporation of the samples under a reducing atmosphere, the internal standard 2-deoxyribose was added. Samples were analyzed by high performance anion exchange chromatography with pulsed amperometric detection (HPAEC-PAD) on a PA-1 column using a Dionex ICS-3000. Neutral monosaccharides were separated by isocratic elution with 2.25 mM NaOH.

EPS determinations were done in duplicate. EPS from planktonic cells were normalized by cell number and EPS from biofilms by the amount of elemental sulfur. Mean values are given with standard deviations (±SD) generally ≤15%.

### ATR-FTIR Spectroscopy

Cell pellets from planktonic cells and EPS samples were spread on a diamond Attenuated Total Reflectance (ATR) apparatus (Pike Technologies, United States) separately attached to a FTIR 430 spectrometer (JASCO, Japan). A blank spectrum was run as a background using the ATR without the biological samples and the baseline shift of the spectra was corrected using the IR solution software (JASCO, Japan). Three biological replicates for each sample were analyzed. At least 64 scans with resolution of 4 cm^-1^ using the Happ-Genzel apodization function were collected for all samples. As all cellular components possess characteristic absorbance frequencies and primary molecular vibrations in between 4000 and 600 wave numbers, the FTIR scan was carried out in this region. Spectral processing was carried out using the IR solution software.

### Image Processing

Fluorescence image analysis and surface coverage calculation were done by using an extended version of the software ImageJ (v1.50i). Maximum intensity projections (MIP) were produced with the software IMARIS version 8.1.2 (Bitplane AG, Zurich, Switzerland).

## Results

### Growth on Elemental Sulfur

**FIGURE 1:**
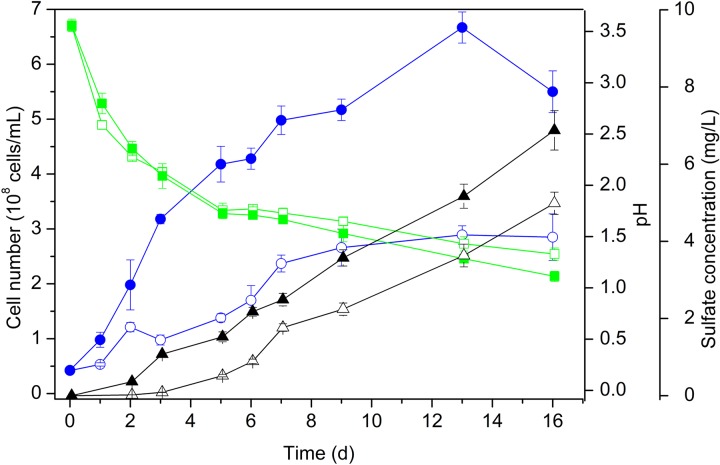
Growth of cells of *Acidianus* sp. DSM 29099 on S^0^ with (solid symbols) and without (open symbols) yeast extract (0.02%). Values of cell number (circle), pH (square), and sulfate concentration (triangle) are shown. Cells were grown with an initial pH 3.5 on Mac medium at 65°C.

When grown on S^0^ as the energy substrate, cell numbers of *Acidianus* sp. DSM 29099 reached approx. 2 × 10^8^ cells/mL (Figure 1). By addition of 0.02% YE cell population increased further to 6 × 10^8^ cells/mL. Cell numbers reached highest values (1.3 × 10^9^ cells/mL) during mixotrophic growth on sulfur and glucose (not shown). Apparently, the addition of YE caused an increase of the growth and of the final stationary level of the cell population (Figure 1). Besides, cells grown on sulfur had a relatively small size (0.5–0.8 μm in diameter) compared to cells grown on sulfur with additional YE (0.7–1.2 μm in diameter) or with YE and glucose (0.8–1.1 μm in diameter, not shown). In both cases, the pH decreased with an increase of the sulfate concentration indicating sulfur oxidation.

### Cell Surface Characterization

#### Surface Analysis by ATR-FTIR

Functional groups like hydroxyl (-OH, 3278 cm^-1^), hydrocarbon (2925 cm^-1^, 1453 cm^-1^), amide I (1637 cm^-1^), amide II (1532 cm^-1^), phosphodiester linkages of RNA or DNA (P = O, 1223 cm^-1^), and polysaccharides (900–1130 cm^-1^) were detected on cell surfaces, if cells were grown on S^0^, PT, or iron(II) sulfate (Supplementary Figure S2). These functional groups indicate that proteins and carbohydrates are dominating on the cell surface of *Acidianus* sp. DSM 29099 and *S. metallicus*^T^.

We also compared the surfaces of S^0^ before and after incubation with cells by ATR-FTIR (Supplementary Figure S3). Results show that sulfur surfaces without cell treatment could not be analyzed well by ATR-FTIR (Supplementary Figure S3, black line). Thus, no functional groups could be assigned. However, on the sulfur surface after incubation with cells similar functional groups as on cell surfaces were detectable, namely, amide (I) and (II) and polysaccharides (Supplementary Figure S3, red line). To clarify whether these compounds on S^0^ surfaces are excreted actively by cells or incidental by physical-chemical adsorption (Marnocha et al., 2019) further studies are needed.

#### Hydrophobicity Test by MATH Using Dodecane

The cell populations interacting with dodecane were always lower than 10% regardless of the growth conditions. In particular, the decrease of the OD values (*n* = 2) was 8 ± 0.9% for S^0^, 6 ± 0.2% for S^0^+YE, and 4.5 ± 0.1% for S^0^+YE+glucose. This indicates that the cells had hydrophilic surfaces.

### Adhesion of Cells to Elemental Sulfur

Cells of *Acidianus* sp. DSM 29099 immediately attached to sulfur surfaces after contact (Supplementary Figure S4). More than 45% of the cells attached to sulfur particles in the presence of YE within 60 min. Around 30% of the cells attached to sulfur with addition of YE and glucose. The adsorption of cells to sulfur reached an equilibrium after 60 min for all conditions tested. In general, addition of YE and glucose reduced the initial cell attachment to S^0^. This might have been due to the reasons that (1), cells favor the use of soluble substrates instead of solid S^0^; (2) glucose and small molecules contained in YE formed a conditioning film on S^0^, which in turn could block cell attachment.

### Biofilm Dynamics of *Acidianus* sp. DSM 29099 on Elemental Sulfur

Various kinds of lectins (>70) had been tested for their binding specificity to the cells of *Acidianus* on S^0^ (Zhang et al., 2015a). Among the lectins binding to cells of *Acidianus* sp. DSM 29099, lectins Con A and AAL were chosen to investigate and monitor biofilm development on S^0^.

#### Biofilm Development of Cells of *Acidianus* sp. DSM 29099 on Elemental Sulfur

Only a few cells of *Acidianus* sp. DSM 29099 attached to S^0^ were visible under AFM or EFM after 5 h of incubation (not shown). This seems to be contradictory to the finding by direct counting that approximately half of the cells did attach to sulfur surfaces (Supplementary Figure S4). This was a result of the available surface area: the total sulfur surface was two times higher for all inoculated cells to attach (not shown). In addition, cells probably attached only transiently and detachment often occurred in the initial attachment stage, as indicated by the presence of microbial footprints on sulfur/pyrite surfaces (see below; Zhang et al., 2019).

**FIGURE 2:**
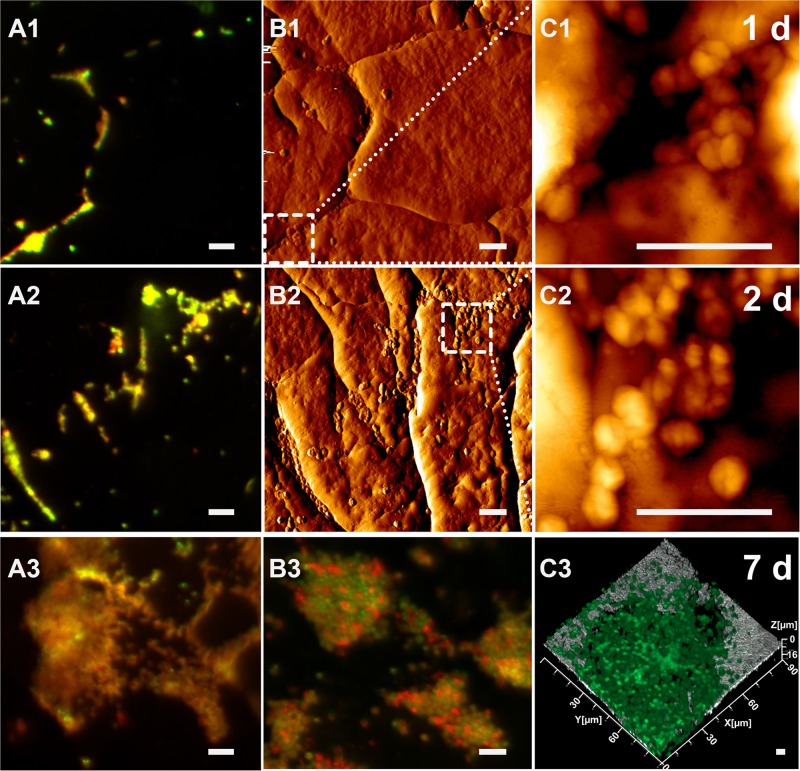
Biofilm development of cells of *Acidianus* sp. DSM 29099 on S^0^ after 1 day (top row), 2 days (middle row), and 7 days (bottom row). **(A1,A2,A3–C2)** EFM images. **(C3)** CLSM image. **(B1,B2)** AFM images corresponding to EFM images **(A1,A2)**, respectively. **(C1,C2)** Enlargement of the frame in panels **(B1,B2)**, respectively. Bars represent 5 μm. Cells were grown on Mac medium with 0.02% YE at 65°C. Cells and EPS were stained by Syto 9 and TRITC-Con A, respectively.

As shown in Figure 2, cells of *Acidianus* colonize a sulfur cube surface after 1 day of incubation. Con A stains glucoconjugates and these indicative of EPS glucoconjugates were cololalized signals from DNA staining by Syto 9 (Figure 2A1–C1). This indicates that mainly capsular EPS were present on cell surfaces during the initial attachment and early stage of microbial-S^0^ interactions for up to 2 days. Corresponding to EFM data, the AFM images showed lobed cells absorbed on the sulfur surface with a diameter of 0.9–1.5 μm, slightly different from the size of planktonic ones (0.8–1.1 μm) visualized on glass slides by AFM. This could be ascribed to morphological variations due to different life styles. The AFM images indicate that cells prefer to attach to surfaces along cracks (Figure 2A2,B2). This colonization pattern was confirmed by the following CLSM studies (Figure 2A3–C3). Besides, big cell clusters were often found located around holes (Figure 2C3 and Supplementary Figure S5). It might be that biofilm cells caused a corrosion of the sulfur surface and developed into depth (indentation of up to 50 μm) during biofilm formation. Further studies are needed to clarify this assumption. During the incubation, the number of cells on the sulfur surfaces increased. The AFM images show that the cells formed a monolayered biofilm after 2 days of incubation (Figure 2A2–C2). Nevertheless, a large part of the sulfur surface area remained free of cells (roughly 95% calculated by ImageJ, not shown). After 7 days of incubation, biofilms covered the sulfur surface significantly (Figure 2A3–C3). Large colonies of cells (up to 30 μm in diameter) became visible. In addition, the EPS signals are not restricted to cells only, but also extend to surrounding areas. Further, the EPS signals from 7 days’ biofilms were stronger than those from the first days (Figure 2A1–A3,B3). All this indicated that cells produced more EPS after attachment for biofilm formation and development.

**FIGURE 3:**
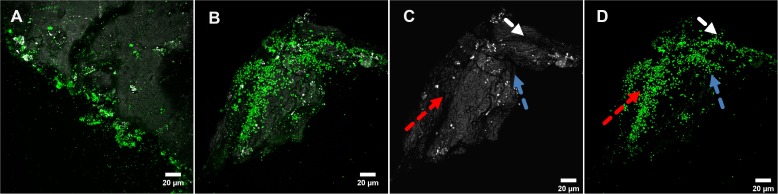
Biofilms of *Acidianus* sp. DSM 29099 grown on sulfur prills **(A,B)**. **(C,D)** are the split channels of panel **(B)**. Arrows in panel **(C)** indicate the surface structure (edge/shielded area) and arrows in panel **(D)** show biofilms developed in these specific areas. Cells were grown on Mac medium with 0.02% YE at 65°C.

A specific attachment and colonization pattern became evident during examination of biofilms on sulfur prills. Cells attached only to the edges of the sulfur prills, regardless of the cracks (Figure 3). Probably due to collisions caused by shaking, cells were mechanically removed. This seemed to indicate that the collisions among sulfur prills caused cell detachment and shaped the biofilm formation and structures.

**FIGURE 4:**
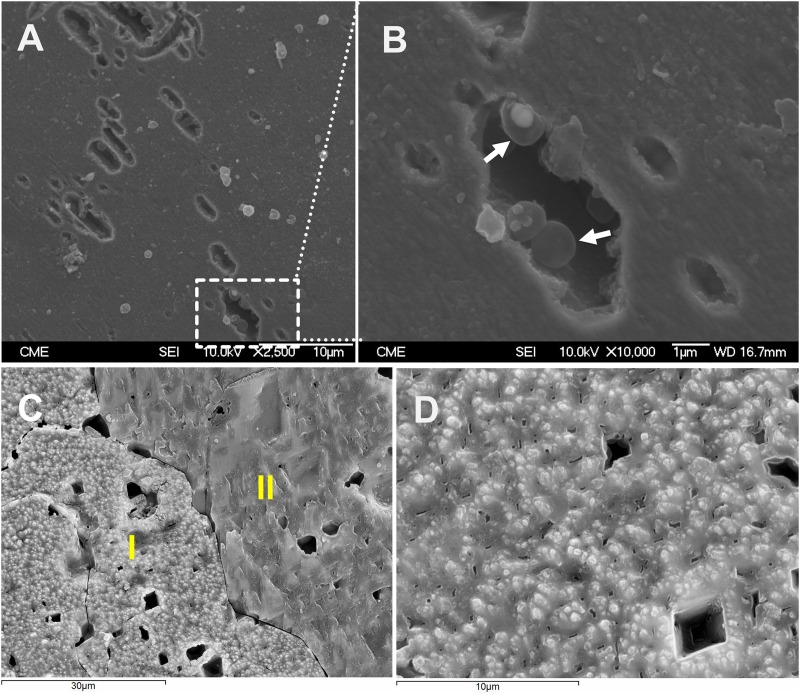
SEM images of biofilm cells attached on S^0^ (**A,B** 3 days; **C,D** 7 days). Image **(A)** shows a dissolution pattern of sulfur by cells of *Acidianus* sp. DSM 29099. Panel **(B)** is enlargement of the frame in panel **(A)**. Arrows in panel **(B)** show cells inside a pit. Panel **(C)** is an overview of a sulfur cube surfaces after 7 days incubation with cells of *Acidianus* sp. DSM 29099. Panel **(D)** shows sulfur surfaces covered by biofilm cells. I and II in panel **(C)** show surfaces free of cells and surfaces covered by biofilm cells, respectively.

**FIGURE 5:**
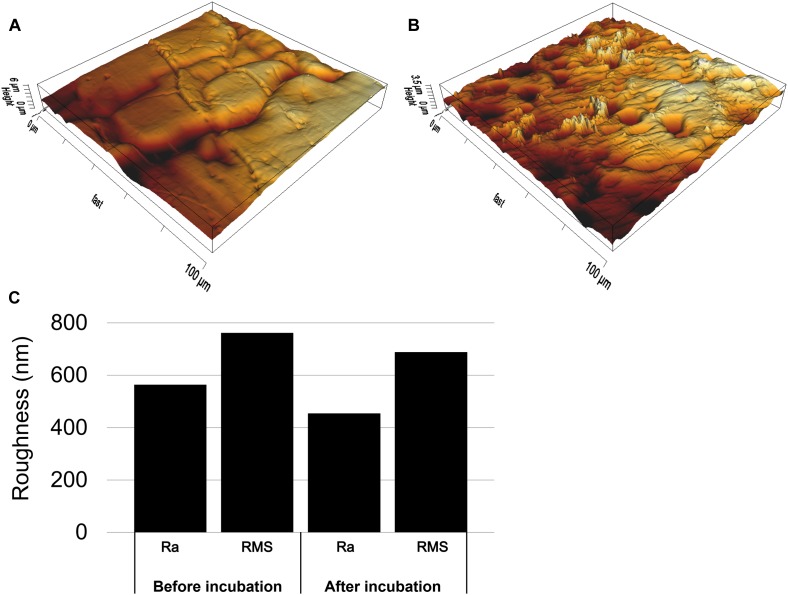
AFM images of sulfur surfaces before **(A)** and after **(B)** incubation with cells of *Acidianus* sp. DSM 29099 and mean surface roughness (Ra: calculated as the Roughness Average and RMS: calculated as the Root Mean Square) of sulfur **(C)** in nm before and after incubation with cells. Cells were incubated in Mac medium with an initial cell number of 5 × 10^8^ cell/mL and pH 3.5 for 7 days.

Scanning electron microscopy was also applied to investigate the distribution of biofilm cells on S^0^. As shown in Figure 4A,B, after 3 days of cultivation, cells were detected on the surfaces. Some of the cells were found sitting inside pits (Supplementary Figure S5), which were distributed heterogeneously over the sulfur surfaces. Biofilm density increased over time and cells were embedded in a matrix of EPS (Figure 4C,D). Figure 4C indicates the heterogeneous distribution of cells on sulfur, maybe due to the different sulfur surface properties. Also, the shaking during cultivation may be a cause of heterogeneous distribution of cells. The AFM images indicate that the sulfur surface is covered by cells and EPS causing a decreased roughness (Figure 5).

### Biofilm Properties

#### Visualization of “Dead Cells” and eDNA in Biofilms Under Fully Hydrated Conditions

**FIGURE 6:**
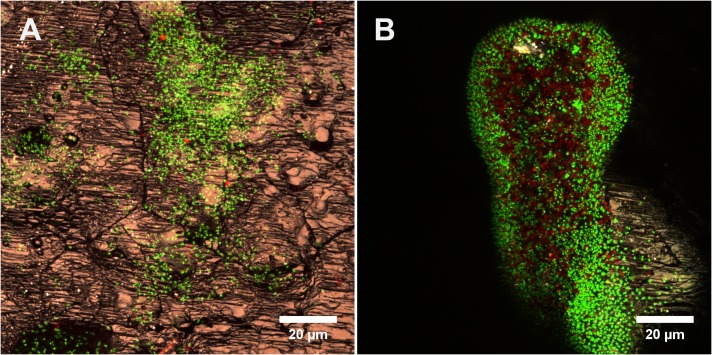
Cells of *Acidianus* sp. DSM 29099 on sulfur coupons stained by Sypro orange (green) and DDAO (red) and visualized by CLSM in fully hydrated conditions. Images **(A)** and **(B)** show monolayer and multilayer biofilms, respectively.

In Figure 6A,B biofilm cells on S^0^ were stained by Sypro and counterstained by DDAO. Interestingly, Sypro stains cells green and DDAO stains large “cells” in red; the latter are probably dead cells/eDNA. In addition, the proportion of DDAO cells increases along with depth (Figure 6B). This observation suggests that cells seem to be distributed differentially along nutrient and oxygen gradients within the biofilm. It has been shown that the decreased availability of oxygen inhibits attachment of *Acidithiobacillus (A.) thiooxidans* to S^0^ (Takakuwa et al., 1979). The dissolved oxygen concentration directly influences the growth of *At. thiooxidans* and its capacity to oxidize S^0^ (Jaworska and Urbanek, 1998). Thus, a lack of dissolved oxygen also may have negative effects on biofilm growth and structures of *Acidianus* sp. DSM 29099.

**FIGURE 7:**
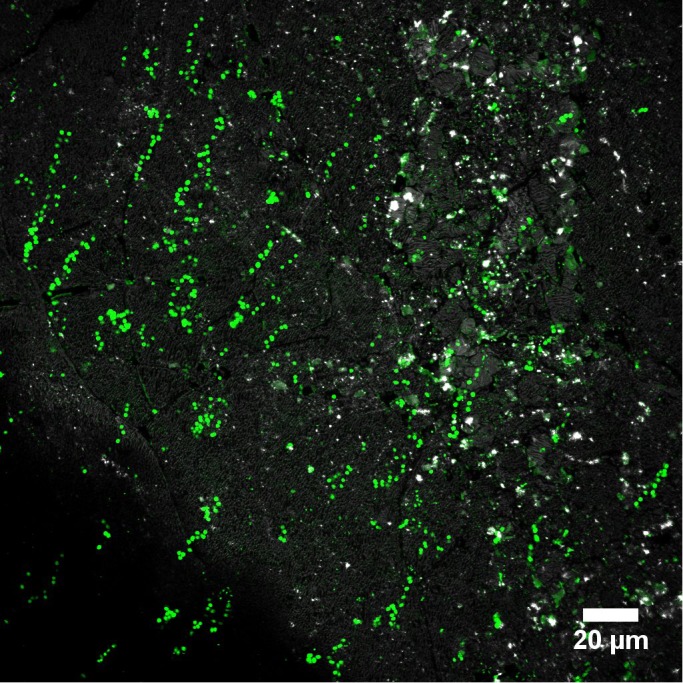
Biofilm cells of *Acidianus* sp. DSM 29099 on S^0^ stained by AAL-A488.

Often, biofilm cells of *Acidianus* were observed to be somehow floating on matrix-compounds or they produced stalks (Figure 7 and Supplementary Movie 1). Cells show a back and forward movement on the sulfur surface. EPS or cellular appendages like stalks or pili mediating cell-S^0^ interactions possibly have sizes in the nano-range (Sullan et al., 2014; van Wolferen et al., 2018). Techniques like nanoscopy (laser based), electron microscopy or scanning tunneling microscopy can be used probably to visualize them (Gorby et al., 2006; Neu and Lawrence, 2014). A lateral movement of cells of *Sulfolobus* on sulfur surfaces has been reported (Weiss, 1973). The nature of real materials allowing the cells to glue themselves to S^0^ awaits further investigation.

#### Observation of Microbial Footprints on the Sulfur Surface

**FIGURE 8:**
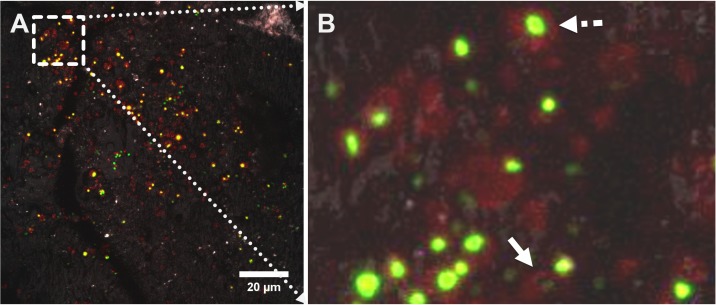
CLSM images showing a maximum projection of an elemental sulfur surface colonized by cells of *Acidianus* sp. DSM 29099 after 1 day of incubation. Cells were double stained with fluorescent FM4-64 (red) and SYBR Green (green). Signals from both fluorescent channels plus light reflection (to image the sulfur surface) were recorded; the merged image from all three channels is shown in panel **(A)**. An enlarged area (selected area in **A**) shows cell footprints **(B)**. An attached cell surrounded by FM4-64 signals and a cell footprint after detachment were indicated by the dashed line and solid line arrows, respectively.

Microbial footprints are adhesive EPS matrix compounds left behind after the detachment of microorganisms from solid interfaces (Neu and Marshall, 1991; Neu, 1992). These may be true adhesive sticky polymers forming the glue in between microbial cells and the interface. As shown in Figure 8, “green dots” representing cells were surrounded mostly by “red dots” representing lipophilic compounds. In many cases only “red dots” devoid of cells are visible, resembling “microbial footprints.” By comparison of attached cells with microbial footprints it can be estimated that approximately 1/3 of the primarily attached cell population had detached to the bulk solution. Detached cells left behind a chemically modified sulfur surface.

### EPS Characterization

#### EPS Analysis by ATR-FTIR

Functional groups corresponding to hydroxyl (-OH, 3396 cm^-1^; 3316 cm^-1^; 3275 cm^-1^), hydrocarbon (∼2929 cm^-1^), amide I (1616 cm^-1^; 1634 cm^-1^), amide II (1389–1536 cm^-1^), carboxylic groups (COO^-^, ∼1394 cm^-1^), and polysaccharides (900–1130 cm^-1^) are present in both types of samples, the capsular and colloidal EPS fractions of *Acidianus* sp. DSM 29099 grown on S^0^ or PT (Supplementary Figure S6). This indicates that proteins and carbohydrates were the main part of the EPS. However, the proportions of the EPS components varied significantly in each ATR-FTIR spectrum, as indicated by the main peaks (Supplementary Figure S6). For instance, in case of colloidal EPS of cells grown on either PT or S^0^, band ratios of polysaccharide/amide I were 2–3. This finding indicates strongly that carbohydrates are the main component of these EPS. The band ratios of polysaccharide/amide I of the capsular EPS of cells grown on either PT or S^0^ were 1–1.3, meaning that both proteins and carbohydrates occurred in similar concentration. The EPS of sessile cells grown on S^0^ exhibited mainly peaks of amide I, carboxylic groups and polysaccharides. These groups had a similar adsorption intensity. Thus, proteins and polysaccharides were produced by growing cells of on S^0^. This was also confirmed by the comparison of the spectra of S^0^ before and after incubation with *Acidianus* sp. DSM 29099 (Supplementary Figure S3).

**Table 1:** EPS composition of *Acidianus* sp. DSM 29099 and *S. metallicus*^T^ grown on RISCs.

Strain	Substrates	EPS type	Sugars	Uronic acids	Proteins	Lipids	DNA	G6PDH
*Acidianus* sp. DSM 29099	K_2_S_4_O_6_	Colloidal^a^	9.8	6.2	2.3	0.1	ND	ND
		Capsular^b^	14.7	0.1	2.7	1.1	ND	ND
	S^0^	Colloidal^a^	40.8	3.1	0.2	0.3	BL	<2%
		Capsular^b^	19.1	0.2	29.6	5.9	BL	<2%
		Sessile^c^	5.2	0.3	1.7	0.6	BL	ND
*S. metallicus*^T^	S^0^	Colloidal^a^	25.9	0.7	2.9	BL	BL	<5%
		Capsular^b^	15.9	0.4	15.7	4.7	BL	<2%
		Sessile^c^	0.5	0.02	8.8	0.2	0.09	ND


*^*a*^mg/L, The unit of content and it indicates the quantity (mg) of EPS per liter culture. ^*b*^mg/g, The quantity (mg) of EPS per gram cell biomass. ^*c*^mg/g, The quantity (mg) of EPS per gram elemental sulfur. BL, below determination; ND, no determination.*

#### EPS Quantification

As shown in Table 1, carbohydrates, uronic acids, proteins, and lipids occurred in the EPS of *Acidianus* sp. DSM 29099. Some activity of G6PDH was detected in the EPS fractions after extraction, which was in general <5% of the activity in cell pellets. The low activity indicated that during the extraction no significant cell lysis occurred (Table 1).

The cells produce comparable amounts of carbohydrates in capsular EPS, whether they were grown on S^0^ or PT, namely 14.7 and 19.1 mg/g, respectively. However, a more than 10-fold increased protein content was measured in capsular EPS of cultures grown on S^0^, compared to the ones grown on PT. In case of the colloidal EPS the carbohydrates were four times more abundant for the S^0^-grown cells than for the PT-grown ones. The total amounts of capsular and colloidal EPS grown on S^0^ were 2.9 and 2.4 times higher than the ones grown on PT, respectively. This is consistent with previous reports showing that bioleaching microorganisms produce an increased amount of EPS, if transferred from soluble to solid substrates (Gehrke et al., 1998; Castro et al., 2014; Li and Sand, 2017; Liu et al., 2018). In the EPS of sessile cells of *Acidianus* sp. DSM 29099 grown with S^0^, 5.2 mg/g carbohydrates, 1.7 mg/g proteins, 0.6 mg/g lipids, and 0.3 mg/g uronic acids were detected. By comparison, the EPS of sessile cells of *S. metallicus*^T^ contained a high content of proteins (8.8 mg/g) and small amounts of DNA (Table 1). In general, the data of the colorimetric determination agreed with the ones of the ATR-FTIR analysis.

**Table 2:** Monosaccharide composition (in %) of EPS from *Acidianus* sp. DSM 29099 and *S. metallicus*^T^ grown on RISCs^∗^.

Strain	Substrates	EPS type	GalNH_2_	Gal	GlcNH_2_	Glc	Xyl	Man
*Acidianus* sp. DSM 29099	K_2_S_4_O_6_	Colloidal	0.0	0.0	0.0	92.7	0.0	7.3
		Capsular	0.8	0.8	4.9	60.6	2.8	30.2
	S^0^	Colloidal	0.0	0.0	0.0	74.4	0.0	25.6
		Capsular	0.9	2.1	0.2	49.8	2.9	44.2
		Sessile	2.5	1.7	0.0	75.0	2.9	17.9
*S. metallicus*^T^	S^0^	Colloidal	0.0	8.7	0.0	63.4	0.0	25.1
		Capsular	0.0	1.0	0.0	50.7	0.0	48.3
		Sessile	0.0	1.3	1.6	58.2	1.3	37.6


*^∗^GalNH_*2*_, galactosamine; Gal, galactose; GlcNH_*2*_, glucosamine; Glc, glucose; Xyl, xylose; Man, mannose.*

The composition of monosaccharides in the EPS of *Acidianus* sp. DSM 29099 and *S. metallicus*^T^ is shown in Table 2. The sugars and sugar derivatives in the EPS of the two thermophilic archaea were glucose, mannose, galactose, xylose, glucosamine, and galactosamine, as detected by HPAEC-PAD. These sugars were also reported to occur with cells of *Acidianus* sp. DSM 29099 and *S. metallicus*^T^ in our previous study using FLBA (Zhang et al., 2015a,b). Fucose was not detected via HPAEC-PAD although it is present in the biofilm matrix as indicated by the positive signals from AAL-A488. The abundance of each sugar varied according to the archaeal species, growth conditions and EPS type and the polysaccharides excreted by the two archaea were heteropolysaccharides. In addition, cells of *Acidianus* sp. DSM 29099 and *S. metallicus*^T^ might excrete more than one type of polysaccharide for the capsular EPS and for the colloidal EPS. Glucose and mannose were the main sugars, accounting for >85% of the sugar content in the EPS. The glucose content was always higher than the one of mannose. Glucose and mannose were the only two sugars detected in the colloidal EPS grown on either PT or S^0^. By contrast, the capsular and the EPS of sessile cells contained additional sugars.

## Discussion

### Attachment of *Acidianus* sp. DSM 29099 to S^0^ and Biofilm Formation

For the thermoacidophilic archaeon *Acidianus* sp. DSM 29099 cells surface properties, biofilm development, and EPS production were investigated. Cell growth of this microorganism (Figure 1; Zhang et al., 2019) was comparable to a closely related but not yet validated species “*Acidianus copahuensis*”, which exhibited physiological versatility and growth flexibility (Giaveno et al., 2013). This implies its roles in biogeochemistry of sulfur and iron cycling and potential use in biomining.

The ATR-FTIR analysis of cell surface agrees with the fact that cells of the order *Sulfolobales* have an S-layer, which is mainly composed of proteins (Albers and Meyer, 2011). According to a previous report (Nichols et al., 1985) the key band ratios correlate with the composition of materials tested by ATR-FTIR. Taking amide (I) (1641 cm^-1^) and polysaccharides (1032 cm^-1^) as main representative peaks, the ratio of protein/carbohydrates for cells grown on iron(II) and S^0^ was 0.7 and 1.0, respectively. It indicates that cells grown on S^0^ produce relatively more surface proteins compared to iron(II) grown cells. Similar findings have been reported for cells of *At. ferrooxidans* (Sharma et al., 2003). Interestingly, the ratio of protein/carbohydrates in the case of *Acidianus* sp. DSM 29099 grown on PT was 1.2. S^0^ is often present as an intermediate product, known as sulfur globules, during microbial oxidation of sulfide and tetrathionate (Schippers et al., 1996; Marnocha et al., 2016). The biogenic sulfur globules produced by cells of *C. tepidum* are enveloped by a layer of proteins and polysaccharides. In addition, these sulfur globules possess reactive surfaces and thus are bioavailable for microbial degradation (Marnocha et al., 2016, 2019). We assume that S^0^ intermediate in the culture of *Acidianus* sp. DSM 29099 grown on PT interacts with cells and such interaction may cause an increased production of cell surface compounds (Supplementary Figure S2).

It has been shown that cell surface properties depend on the type of substrates for growth (soluble or insoluble). Cells of *At. ferrooxidans* on sulfur, pyrite, and chalcopyrite exhibited greater hydrophobicity than iron(II)-grown cells (Devasia et al., 1993). Our hydrophobicity tests indicate that *Acidianus* sp. DSM 29099 cell have a hydrophilic surface, although their energy substrate S^0^ rather argues for its hydrophobicity. A similar result was reported for cells of *A. manzaensis* grown on S^0^ (He et al., 2008). FTIR spectroscopy of sulfur-grown cells of *At. ferrooxidans* showed a proteinaceous new cell surface appendage of mineral-grown cells, which is probably responsible for adhesion to the mineral substrates (Devasia et al., 1993). Zinc and copper ions adapted cells of *At. ferrooxidans* produced more EPS with a modified composition compared to non-adapted iron(II)-grown cells. Also, cell hydrophilic properties increased after adaptation (Vilinska and Rao, 2011). Functional groups representing carbohydrates, proteins and lipids on cell surface of *At. ferrooxidans* were detected by ATR-FTIR (Li et al., 2016). Biofilm cells of *S. acidocaldarius* and *S. solfataricus* showed increased carbohydrate functional groups in their FTIR spectra compared to planktonic cells (Koerdt et al., 2011). More proteins were detected on the cell surface of *A. manzaensis* grown on chalcopyrite and S^0^ than on iron(II) ions (He et al., 2008). In the current study, the analyses of the *Acidianus* sp. DSM 29099 cell surface as well as S^0^ surface components by ATR-FTIR indicated peaks representing proteins and carbohydrates on the cell surface (Supplementary Figure S2), whereas surfaces of S^0^ incubated with cells were covered by these microbial surface compounds (Supplementary Figure S3). The increased protein content (as revealed by protein/carbohydrate ratio) in surface components for cells grown on S^0^ and PT compared to iron-grown cells indicated that *Acidianus* sp. DSM 29099 (Supplementary Figure S2) responds to substrates in a similar way as bacteria do.

Extracellular polymeric substances contain functional groups, e.g., carboxyl and phosphate groups. These are usually ionized and negatively charged, resulting in a hydrophilic surface (Flemming and Wingender, 2010). It seems that cells of *Acidianus* sp. DSM 29099 adhered to S^0^ and modified S^0^ in order to utilize insoluble sulfur. Also, microbial interactions with S^0^ caused a decrease of S^0^ surface roughness (Figure 5). This change is probably due to the formation of a biofilm and EPS (Li and Sand, 2017). Nevertheless, further analysis is needed to verify this assumption of active microbial excretion of organic compounds on the S^0^ surfaces, e.g., by Raman spectroscopy, as it is suitable and has been successfully used for analysis of S^0^ and S^0^ covered with biofilms and an EPS matrix (Marnocha et al., 2019).

A biofilm cycle generally includes initial attachment, irreversible attachment, biofilm maturation, and detachment. The biofilm formation is a consequence of physical interactions of microorganisms and their EPS or other attachment factors with a surface (Bellenberg et al., 2015). Several factors like temperature, pH, agitation, and medium ionic strength influence microbial attachment and subsequent biofilm development. The effects of shear force due to shaking has been shown for three *Acidithiobacillus* species attached to pyrite (Bellenberg et al., 2015). Exclusively, surface fissures, pores, or defect sites are the sites to be colonized, if incubated with agitation. In contrast, pyrite (grains) colonization under static conditions occurs homogeneously, and the colonization pattern is independent of surface defect sites (Bellenberg et al., 2015). For cells of *Acidianus* sp. DSM 29099 shaking seemed to contribute to a special colonization pattern and biofilm morphology, i.e., cell attachment to edges of the sulfur prills (Figure 3).

Attachment patterns of acidophilic bacteria to their solid energy substrates such as pyrite or S^0^ have been observed. Attachment sites of the sulfur-oxidizer *At. caldus* to pyrite, marcasite and arsenopyrite coincide with dissolution pit edges and secondary sulfur minerals, which are optimal sites for chemoautotrophic growth on sulfur (i.e., the mineral surface) (Edwards et al., 2000a). Preferential attachment of *At. ferrooxidans* (Mangold et al., 2008), *Leptospirillum (L.) ferriphilum* (Noël et al., 2010) to microcracks/imperfections/defects on pyrite surfaces was also reported. Cells of moderate thermophilic archaeon *Ferroplasma acidiphilum* showed similar behavior like *At. ferrooxidans* and *L. ferriphilum* (Zhang et al., 2014). In a study of ancient microbe-mineral surface interactions it was speculated that original iron(III)-reducing bacteria may preferentially attach to structural imperfections on hematite surfaces (Kolo et al., 2009). So far, this colonization behavior on S^0^ or pyrite seems to be similar for the reported leaching bacteria and archaea (Zhang et al., 2016). We also observed that cells of *Acidianus* sp. DSM 29099 preferentially attach to surfaces along cracks (Figure 2A2,B2). A mobility assay performed on a semisolid agar plate indicated that cells of *Acidianus* sp. DSM 29099 did not show significant swarming ability (not shown). To address whether attachment of *Acidianus* sp. DSM 29099 is via cell appendages and driven by energy sensing genetic systems or physical-chemical factors, further investigations are needed.

Several studies on microbial interactions with sulfur and the visualization of biofilms formed by acidophiles on S^0^ revealed that cells form monolayers, which are different from the biofilms with multilayers of cells formed by *Acidianus* sp. DSM 29099 (e.g., Figure 2C3). However, similar as we detected in the current study, the corrosion sites associated with cells of other species are also evenly distributed over the S^0^ surface, e.g., for *At. thiooxidans* (Schaeffer et al., 1963), *Sulfolobus* sp. (Weiss, 1973), *Thiobacillus denitrificans* (Baldensperger et al., 1974), and *At. ferrooxidans* (Espejo and Romero, 1987).

Although extracellular DNA is thought to play important roles in attachment and biofilm formation of microorganisms (Whitchurch et al., 2002), only few reports are available for bioleaching microbes in this respect. The presence of eDNA in acidophilic microorganisms such as *S. metallicus*^T^ (Zhang et al., 2015b), *Sulfobacillus (Sb.) thermosulfidooxidans* ST (Yu et al., 2018), and *A. manzaensis* (Liu et al., 2018) indicate its role in mediating interactions in bioleaching environments. In the current study, we used a DDAO stain which should not penetrate cell membranes and is commonly used to stain extracellular DNA and cells without intact cell membranes in biofilms (Koerdt et al., 2010). The use of DDAO allowed us to identify the “dead cells”/eDNA of biofilm cells of *Acidianus* sp. DSM 29099. The functions of eDNA in biofilm formation and sulfur metabolism await further investigation.

During interactions of cells with S^0^ transit attachment was observed as indicated by the presence of microbial footprints (Figure 8). Such transit attachment to solid S^0^ was also reported for cells of *C. tepidum* (Hanson et al., 2016). This form of cell-S^0^ interaction was regarded as a contribution to a syntrophic sulfur cycle (sulfides, globule S^0^ and polysulfides), from which cells of *C. tepidum* benefit (Marnocha et al., 2016).

The observations of such footprint material have been reported for a mixed culture of mesophilic acidophilic chemolithotrophs (*Leptospirillum ferriphilum* was predominant) on sphalerite ore particles (Ghorbani et al., 2012), for *At. ferrooxidans* on pyrite (Mangold et al., 2008), and for *At. ferrooxidans* on sulfur (Rojas et al., 1995). The factors causing cell detachment could be (1) not sufficient electrochemical attraction/hydrophobic interactions between cell and S^0^ surface (Li et al., 2019), (2) chemotaxis to soluble substrates (Tan and Chen, 2012), (3) mechanical shear forces from particles and fluids, (4) genetically controlled systems (Díaz et al., 2018). In summary, the results indicate that extracellular polymers like lipids are most likely interacting with sulfur. It has been reported that acidophilic bacteria like *At. thiooxidans* and *At. ferrooxidans* are able to produce phospholipids as wetting agents (Beebe and Umbreit, 1971; Arredondo et al., 1994), which facilitate oxidation of S^0^ by decreasing the surface tension between the sulfur particle and the liquid medium. Also, for the EPS of *At. ferrooxidans* lipids are important compounds for growth on pyrite or S^0^ (Gehrke et al., 1998; Harneit et al., 2006). For cells of *Sulfolobus* sp. and *Thiobacillus albertis* pili and cell wall components like a glycocalyx were found to be involved in the connection with S^0^ (Bryant et al., 1983, 1984; Laishley et al., 1986). The presence of mucous polysaccharides in the EPS was visualized by ruthenium red staining (Bryant et al., 1983). Additionally, membrane blebs occurred and these are hypothesized to aid the cells in colonization (Crescenzi et al., 2006) as well as overcoming the hydrophobic barrier necessary for their growth on S^0^ (Knickerbocker et al., 2000).

### EPS Production

Extracellular polymeric substances production and composition depends on the type of substrate, on which the cells grow. The EPS of cells of a thermophilic sulfur- and iron-oxidizer *A. manzaensis* YN-25 contain proteins, polysaccharides and a small amount of eDNA (Liu et al., 2018). In addition, cells adapted to chalcopyrite, pyrite and S^0^ secrete more EPS than iron(II)-adapted-cells, while the EPS of iron(II)-adapted-cells have the highest protein content, and the EPS of S^0^ adapted cells have the highest content of lipids (Liu et al., 2018). EPS produced by cells of *At. ferrooxidans* mainly consist of common carbohydrates and lipids. In addition, pyrite-grown cells have a more than 10-fold amount of EPS, if compared to iron(II)-grown cells (Gehrke et al., 1998; Harneit et al., 2006). For cells of *L. ferrooxidans*^T^ the total amount of EPS increases and the EPS composition changes with increased concentrations of galactose and ferrous iron. In addition, cells pre-cultured on galactose produce “stickier” EPS than the ones on iron(II) (Aguirre et al., 2018). Cells of *Sb. thermosulfidooxidans* excrete a substantial amount of humic acids apart from proteins and carbohydrates (Li and Sand, 2017). For mixed cultures of mesophiles, moderate thermophiles, and thermophiles growing on pyrite, sphalerite and chalcopyrite, 70% of the EPS extracted are composed of carbohydrates and a small amount of proteins with trace levels of humic and uronic acids (Govender and Gericke, 2011). A characterization of two acidophilic microbial biofilms from Iron Mountain (CA, United States) indicates that EPS are composed of carbohydrates, metals, proteins and minor quantities of DNA and lipids (Jiao et al., 2010).

The extracellular polysaccharides identified in the EPS of *Sulfolobus* and the haloarchaea *Halobacterium*, *Haloferax*, and *Halorubrum* are mannose, galactose, glucose, *N*-acetylglucosamine, and/or *N*-acetylneuraminic acid (Nicolaus et al., 1993; Koerdt et al., 2010; Fröls et al., 2012; Fröls, 2013). The EPS polysaccharides from the hypothermophile *Thermococcus litoralis* contain mannose as the only monosaccharidic constituent (Rinker and Kelly, 1996). It must be pointed out that these archaea are usually grown on organic substrates, which are not comparable to the current study using inorganic S^0^. However, it seems that sugar monomers like glucose and mannose are often constituent monosaccharides of the EPS polysaccharides (Table 2; Nicolaus et al., 1993; Koerdt et al., 2010; Fröls et al., 2012). It has been reported that the bioleaching bacterium *At. ferrooxidans* produces rhamnose, fucose, xylose, mannose and glucose, if grown on ferrous iron and pyrite (Gehrke et al., 1998; Harneit et al., 2006). This explains, why the lectin Con A, which specifically binds a-glucopyranosyl and a-mannopyranosyl residues (So and Goldstein, 1968), stains not only bioleaching archaea or bacteria but also other species in general (Staudt et al., 2003; Koerdt et al., 2010; Bellenberg et al., 2012; Zhang et al., 2014). Still, whether a common compound exists in the EPS of microorganisms is unknown and needs further investigation (Neu and Lawrence, 2016).

**FIGURE 9:**
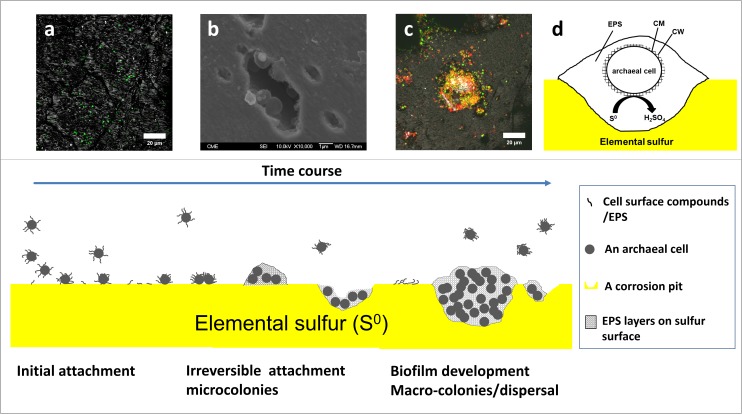
The model of biofilm formation of *Acidianus* sp. DSM 29099 on S^0^. Cells undergo several states including intial adhesion, irreversible attachment, biofilm development, and disperal. **(a,b,c)** are CLSM and SEM images accompanying the first three statuses, respectively. Panel **(d)** shows that an archeal cell is embedded in a large extracellular polymeric substances (EPS) matrix. EPS, extracellular polymeric substances; CM, cell membrane; CW, cell wall.

To summarize, the formation of a biofilm by *Acidianus* sp. DSM 29099 is illustrated in Figure 9. Planktonic cells get in contact with the sulfur surface and attach to S^0^ reversibly and then firmly. EPS compounds like polysaccharides containing glucose and mannose, proteins and lipids are mediating the initial cell-S^0^ interactions. The S^0^ surfaces become covered by the excreted EPS. In the second step, firmly attached cells excrete EPS and start to multiply. Microcolonies together with corrosion pits can be seen. In the maturation step, macrocolonies and multilayers of biofilms are formed, and cells are embedded and held together in an EPS matrix. Biofilms are formed with a thickness of 10–100 μm in diameter.

## Conclusion and Significance

This work aims at a better understanding of the dynamic process of archaea-sulfur interfacial interactions. The biofilms and micro-/macro-colonies of cells of *Acidianus* sp. DSM 29099 on the sulfur surface comprise an extracellular matrix. This matrix contains carbohydrates like glucose and mannose, proteins, and lipids. The EPS matrix mediates and enhances interactions between cells and S^0^ and may extend the reaction spaces at the microbial-substrate interfaces (Vera et al., 2013; Marnocha et al., 2019). EPS components like lipids and/or lipophilic amino acids mediate the initial attachment of cells to S^0^. Sugars like glucose, mannose and fucose are important for a colonization of sulfur and a mature biofilm architecture of acidophilic thermophiles. Future work shall focus on the underlying molecular mechanisms (genetic regulations) controling the attachment and detachment processes and the EPS functionality in binding and possibly transporting elemental sulfur.

## Author Contributions

RZ and WS designed the experiments. RZ and TN performed the CLSM experiments and analyzed the data. QL performed the AFM experiments and analyzed the data. RZ, YZ, and VB performed the experiments of EPS analysis. RZ, AS, and WS prepared the manuscript.

## Conflict of Interest Statement

The authors declare that the research was conducted in the absence of any commercial or financial relationships that could be construed as a potential conflict of interest.
