# Supplementary material for: Insight Into Interactions of Thermoacidophilic Archaea With Elemental Sulfur: Biofilm Dynamics and EPS Analysis
Source: Front Microbiol. 2019 May 10;10:896. doi: 10.3389/fmicb.2019.00896 (PMC6524610; doi:10.3389/fmicb.2019.00896)
Supplement: Supplementary file 1 [file Data_Sheet_1.docx]

**Supplementary data**

**Insight into interactions of thermoacidophilic archaea with elemental sulfur: biofilm dynamics and EPS analysis**

Ruiyong Zhang^1,5^, Thomas R. Neu^2^, Qian Li^3^, Véronique Blanchard^4^, Yutong Zhang^5^, Axel Schippers^1^, Wolfgang Sand^*5,6,7^

^1^ Federal Institute for Geosciences and Natural Resources (BGR), 30655 Hannover, Germany

^2^ River Ecology, Helmholtz Centre for Environmental Research-UFZ, Magdeburg, Germany

^3^ Key Laboratory for Water Quality and Conservation of the Pearl River Delta, Ministry of Education; School of Environmental Science and Engineering, Guangzhou University, Guangzhou 510006, China

^4^ Institute of Laboratory Medicine, Charité Medical University, Berlin, Germany

^5^ Biofilm Centre, Universität Duisburg-Essen, Essen, Germany

^6^ College of Environmental Science and Engineering, Donghua University, Shanghai, China

^7^ TU Bergakademie Freiberg, Freiberg, Germany

* *Correspondence*:

[wolfgang.sand@uni-due.de](mailto:wolfgang.sand@uni-due.de)


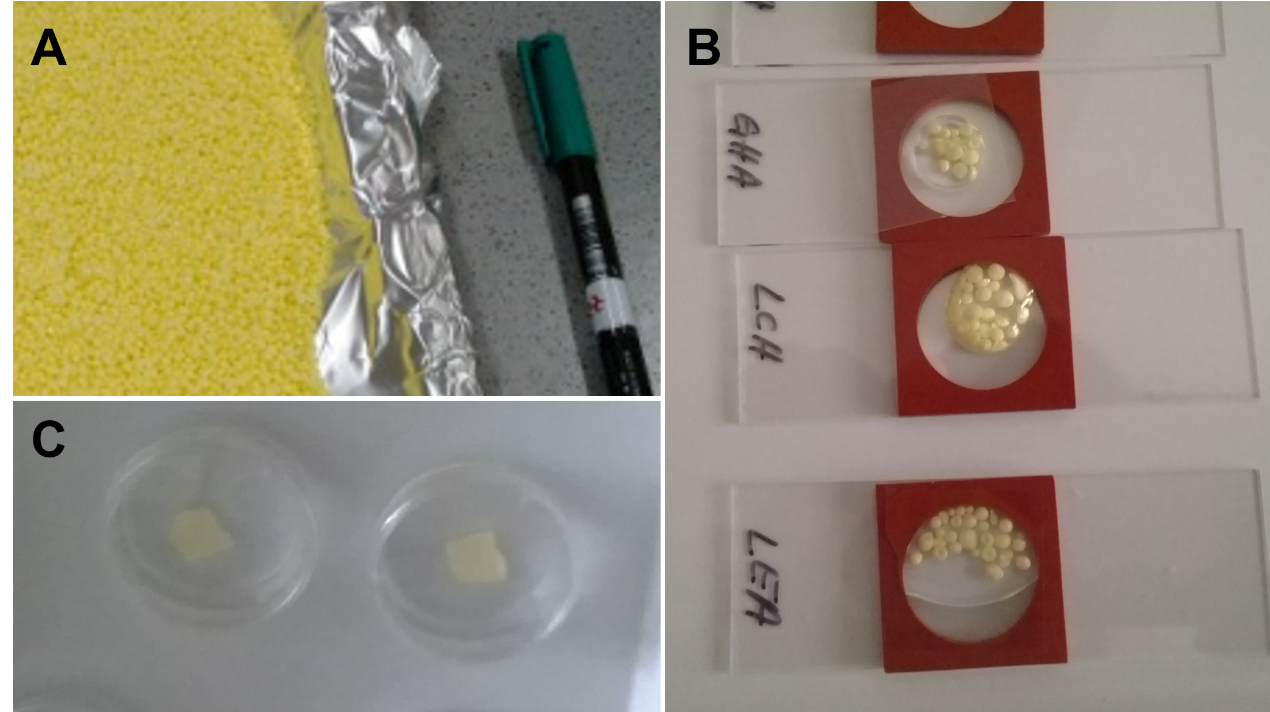


**Supplementary Fig. S1.** The sulfur prills (A) and sulfur cubes used in this study. B and C are examples of sample staining for biofilms on sulfur prills and sulfur cubes, respectively.


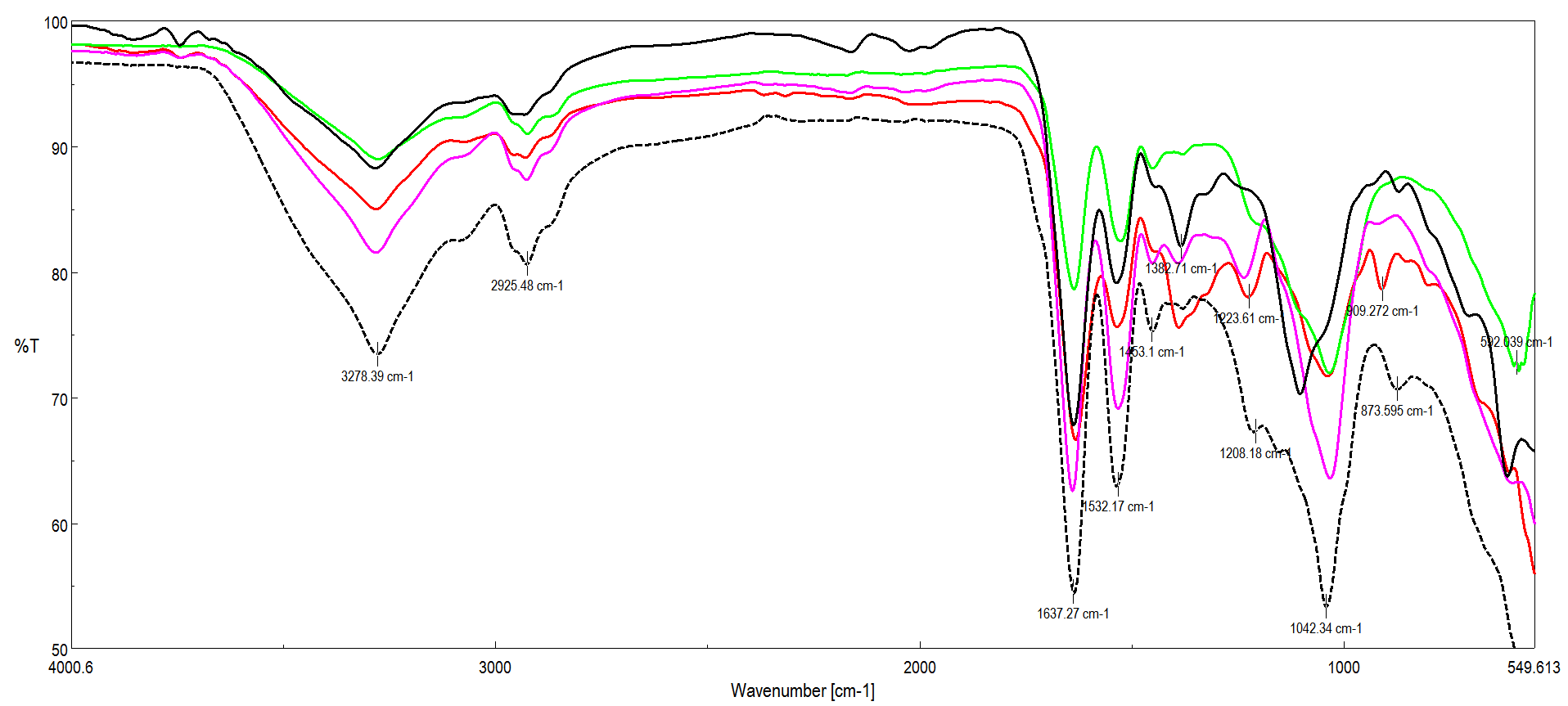


**Supplementary Fig. S2.** Overlay spectra ATR-FTIR data of *Acidianus* sp. DSM 29099 grown on potassium tetrathionate (PT) + YE (red), iron(II) sulfate (green), S^0^ (pink) and S^0^ + YE (black); *S. metallicus*^T^ grown on S^0^ + YE (black, dash).


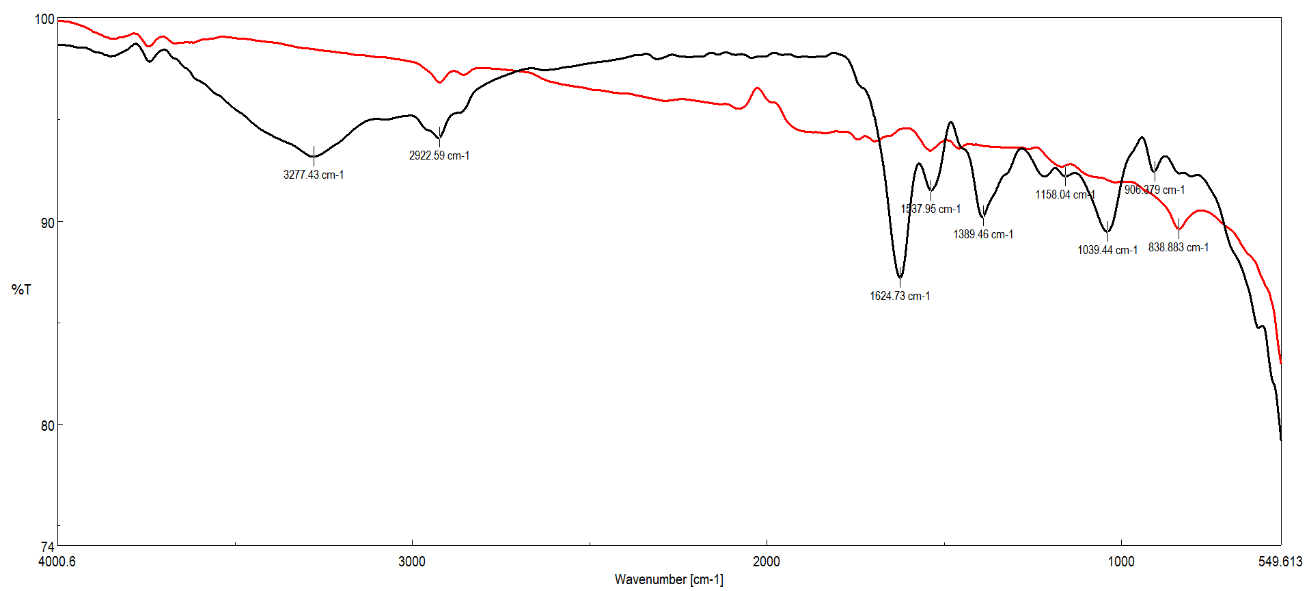


**Supplementary Fig. S3**. Overlay ATR-FTIR spectra of S^0^ before (red) and after (black) incubation with cells of *Acidianus* sp. DSM 29099

**

**

**Supplementary Fig. S4**. Attachment behavior of *Acidianus* sp. DSM 29099 to S^0^. Attachment experiments were carried out in 50 mL Mac media containing 2.0×10^8^ cells/mL and 10 g S^0^ on a rotary shaker (120 rpm) at 65 °C. The number of attached cells was calculated by subtraction of the remaining planktonic cells from the number of cells inoculated. Average values from three parallels are shown and error bars represent the standard derivations.


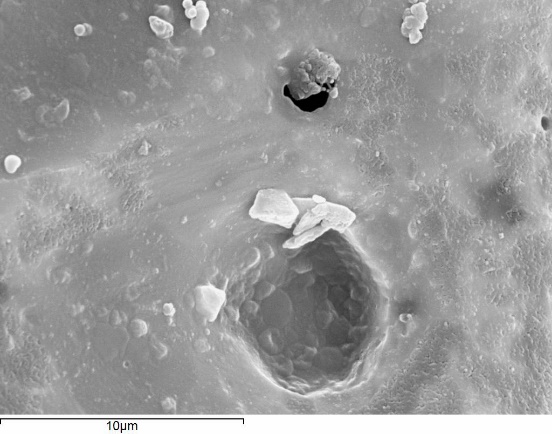


**Supplementary Fig. S5**. Cells were imbedded in EPS matrix and were associated with holes/pits on sulfur surface and possibly ‘ate’ the sulfur resulting the formation of “corrosion pits”


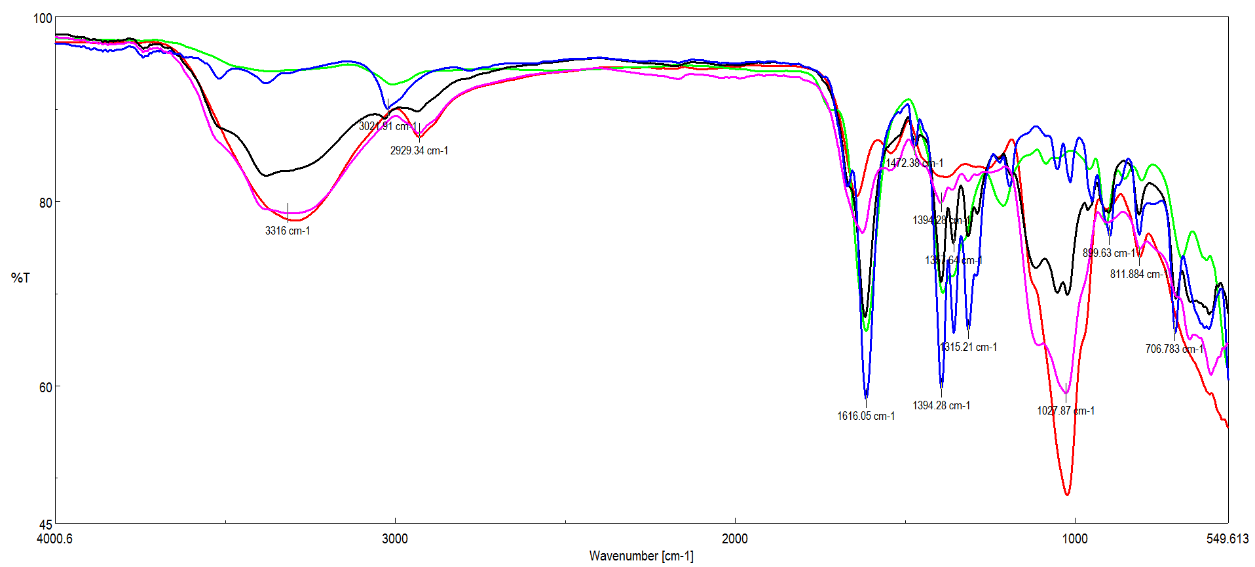


**Supplementary Fig. S6.** Overlay spectra ATR-FTIR data of colloidal EPS (red), capsular EPS (green) and sessile EPS (black) of *Acidianus* sp. DSM 29099 grown on S^0^; colloidal EPS (pink) and capsular EPS (blue) of *Acidianus* sp. DSM 29099 cells grown on PT

**
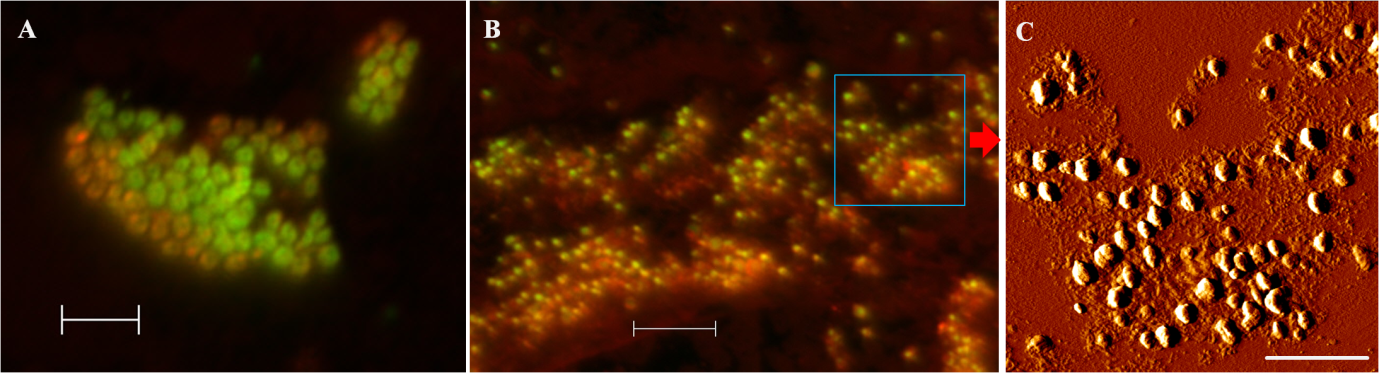
**

**Supplementary Fig. S7**. Comparison of planktonic cells of *Acidianus* sp. DSM 29099 grown on S^0^ (7 days) before (A) and after treatment by 20 mM EDTA. Cells were fixed on glass slides and stained by TRITC-conjugated Con A and Syto 9, respectively. Samples were visualized by EFM (A and B) combined AFM (C). Bars represent 5 µm

Supplementary Movie 1. Biofilm cells stained by Syto 9 seem to float on gel-like compounds or they have stalks.
