# Supplementary figures and images for: Insight Into Interactions of Thermoacidophilic Archaea With Elemental Sulfur: Biofilm Dynamics and EPS Analysis
Source: Front Microbiol. 2019 May 10;10:896. doi: 10.3389/fmicb.2019.00896 (PMC6524610; doi:10.3389/fmicb.2019.00896)

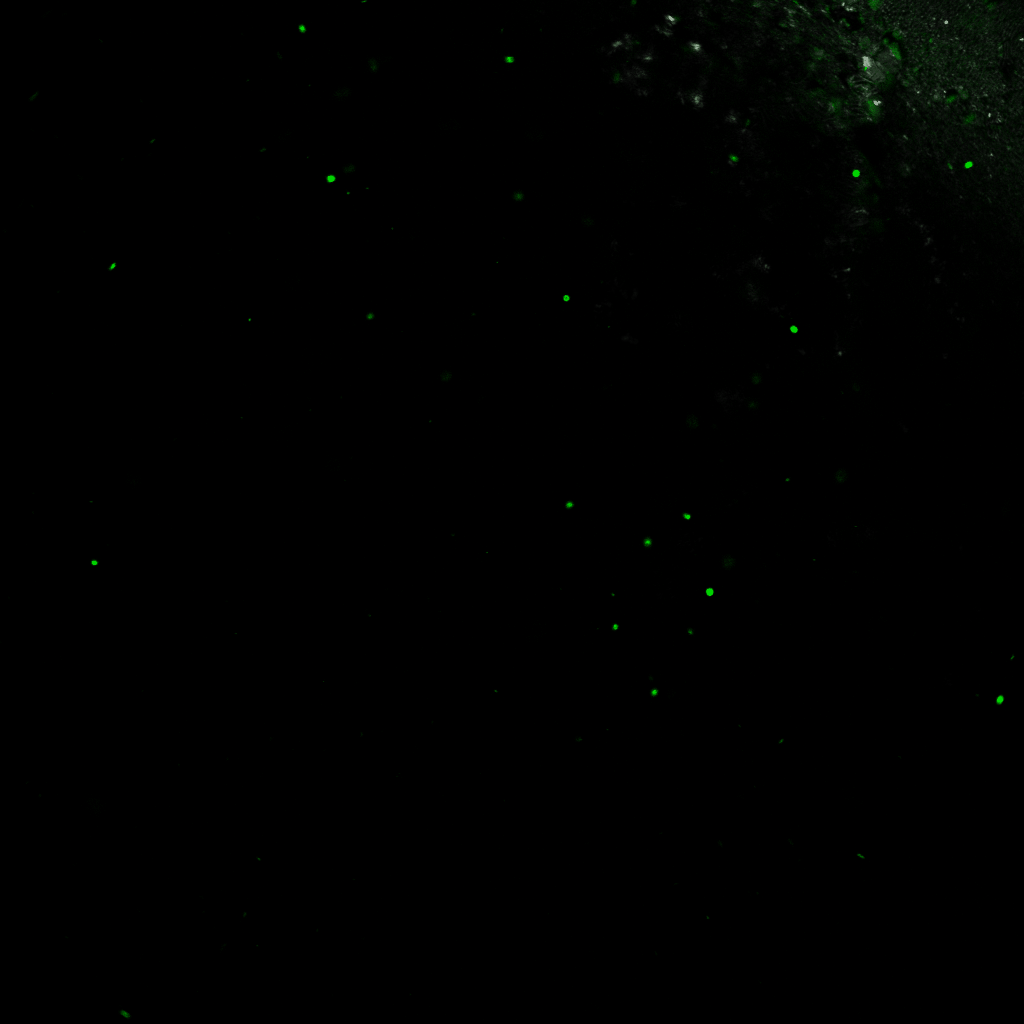

Supplement: Supplementary file 2 [file Data_Sheet_2.zip › Supplementary Movie 1.gif]
